# The Urban Heat Island and its spatial scale dependent impact on survival and development in butterflies of different thermal sensitivity

**DOI:** 10.1002/ece3.2166

**Published:** 2016-05-21

**Authors:** Aurélien Kaiser, Thomas Merckx, Hans Van Dyck

**Affiliations:** ^1^ Behavioural Ecology and Conservation GroupBiodiversity Research CentreEarth and Life InstituteUniversité catholique de Louvain (UCL)Louvain‐la‐NeuveBelgium

**Keywords:** Developmental plasticity, larval survival, Lepidoptera, microclimate, spatial scale, urbanization

## Abstract

Climate alteration is one of the most cited ecological consequences of urbanization. However, the magnitude of this impact is likely to vary with spatial scale. We investigated how this alteration affects the biological fitness of insects, which are especially sensitive to ambient conditions and well‐suited organisms to study urbanization‐related changes in phenotypic traits. We monitored temperature and relative air humidity in wooded sites characterized by different levels of urbanization in the surroundings. Using a split‐brood design experiment, we investigated the effect of urbanization at the local (i.e., 200 × 200 m) and landscape (i.e., 3 × 3 km) scale on two key traits of biological fitness in two closely related butterfly species that differ in thermal sensitivity. In line with the Urban Heat Island concept, urbanization led to a 1°C increase in daytime temperature and an 8% decrease in daytime relative humidity at the local scale. The thermophilous species *Lasiommata megera* responded at the local scale: larval survival increased twofold in urban compared to rural sites. Urbanized sites tended to produce bigger adults, although this was the case for males only. In the woodland species *Pararge aegeria*, which has recently expanded its ecological niche, we did not observe such a response, neither at the local, nor at the landscape scale. These results demonstrate interspecific differences in urbanization‐related phenotypic plasticity and larval survival. We discuss larval pre‐adaptations in species of different ecological profiles to urban conditions. Our results also highlight the significance of considering fine‐grained spatial scales in urban ecology.

## Introduction

In the current era of global change, urbanization has recently drawn the attention of evolutionary ecologists (Shochat et al. 2006; Alberti 2015). While urbanization consists of multiple, partly interrelated drivers, one of the most widely cited ecological effects of urbanization is the Urban Heat Island (UHI) effect (Oke 1982; Arnfield 2003). Cities typically experience higher temperatures due to the elevated prevalence of heat‐absorbing and emitting surfaces and additional heat production, compared to rural areas. With increasing urbanization, impervious surfaces become the predominant land cover, leading to increased water run‐off and reduced evapotranspiration (Arnold and Gibbons 1996). These alterations to the urban water cycle, together with increased ambient temperatures, cause significant drops in relative air humidity of strongly urbanized areas (e.g., Unkasevic et al. 2001). Hence, urbanization provides an interesting set‐up for testing predictions on life‐history theory and thermal evolutionary ecology by means of in situ developmental experiments along urbanization gradients.

The spatial and temporal distribution of most species is ultimately climate‐driven (Sexton et al. 2009). Temperature and humidity are key climatic variables affecting important life‐history processes (e.g., survival and phenotype development). In general, species are characterized by certain optima for temperature and humidity; moving away from these optima will lower survival. Yet, temperature and humidity interact determining the conditions under which individuals manage to reach the reproductive stage (e.g., Krasnov et al. 2001). Both factors are important in regulating the expression of phenotypic traits. This holds true especially for ectotherms, which are directly affected by ambient temperature for many aspects of their life. For instance, several studies on *Drosophila* flies have shown the importance of temperature in shaping physiology, life‐history traits and morphology (Partridge et al. 1995; Crill et al. 1996; Frazier et al. 2008). Most ectotherms mature at smaller size when forced to develop at higher temperatures, as a consequence of shortened development (i.e., temperature‐size rule; Atkinson 1994). This temperature‐mediated modification of body size is highly relevant, given the link between body size and several fitness components, including fecundity, access to mates and survival (Kingsolver and Huey 2008). Besides the direct effect on ectotherms, elevated temperature may also indirectly affect insect herbivores via modification of host‐plant quality (Zvereva and Kozlov 2006).

Biological processes may not only depend on local conditions (i.e., within the habitat patch), but may also vary with the ecological context at larger spatial scales (Thies et al. 2003). The spatial scale at which organisms perceive and interact with their environment may relate to particular ecological and life‐history traits. For instance, mobile species are largely affected by processes acting at wider scales compared to more sedentary species (Oliver et al. 2010; Raebel et al. 2012). In general, small invertebrates are likely to be influenced by climatic conditions prevailing at finer scales than larger‐sized taxa, such as birds and mammals. Recent work has revealed that temperatures within particular habitats may deviate strongly from average values at the regional scale (Suggitt et al. 2011; Scheffers et al. 2014). Hence, although urban areas may overall be warmer and drier than surrounding areas, it is likely that local habitat types within urban areas interact with the urban, landscape‐scale climate. As a result, specific vegetation types may locally mitigate the broad‐scale effect of urban warming, contributing to a mosaic of sites with different microclimatic characteristics at the landscape scale.

We are interested in urbanization‐related changes in regional climates and in local microclimates that may affect survival and phenotypic trait expression of ectotherms. Insects in general, and butterflies in particular, have been popular study systems for addressing basic and applied questions regarding developmental plasticity relative to environmental variation, including variation in temperature (e.g., Fischer and Karl 2010). Here, we tested (1) whether the UHI concept holds for butterflies and their larval microhabitats, and (2) whether urbanization‐mediated effects on microhabitats/microclimates (as opposed to a larger spatial scale) affect the biological fitness of these ectothermic organisms.

Specifically, we addressed inter‐ and intraspecific variation in biological fitness along an urbanization gradient in a temperate‐zone area using a split‐brood design experiment. We did so by recording larval survival and adult body mass, and we considered urbanization at both a coarse (3 × 3 km plots) and a fine (200 × 200 m subplots) spatial scale. We made use of two closely related satyrine butterfly species that differ in thermal ecology. *Pararge aegeria* is a shade‐tolerant forest species whereas *Lasiommata megera* is a thermophilous grassland species. Moreover, in NW‐Europe, *P. aegeria* evolved an open landscape ecotype that occurs outside woodland in agricultural landscapes along hedgerows (Merckx et al. 2003). Together, the two *P. aegeria* ecotypes (woodland and agricultural ecotype) and *L. megera* represent a gradient from closed, cool, and humid environmental preferences to open, warmer, drier, and more variable environmental conditions.

As observed in most ectotherms, butterflies reach a smaller size at reproduction when they develop under high temperatures (e.g., Karl and Fischer 2008). As a consequence, we expect adult butterflies to reach a lower body mass under urban conditions compared to rural conditions due to the UHI effect. Both direct effects (i.e., on the butterfly) and indirect effects (i.e., through host‐plant quality modification) of temperature may contribute to this pattern. We also predict the agricultural ecotype of *P. aegeria* to display a higher level of developmental plasticity (i.e., steeper reaction norms) compared to the woodland ecotype (Vandewoestijne and Van Dyck 2011). However, taking into account the thermal ecology of each species and ecotype of *P. aegeria*, different predictions can be made. The performance of butterfly species from open, dry habitats generally peaks at a higher temperature than for woodland species (Karlsson and Wiklund 2005). Under this scenario, we predict larvae of the thermophilous grassland species *L. megera* to survive better, and to reach a larger adult body mass in urban areas compared to rural areas. Since woodland populations of *P. aegeria* perform better at low temperatures than do agricultural populations, with the reverse pattern occurring at higher ambient temperature (Karlsson and Van Dyck 2005), we predict individuals of a woodland origin to perform better (i.e., increased larval survival and adult body mass) at sites with low levels of urbanization (i.e., cooler and more humid conditions), whereas we predict the agricultural ecotype to be advantaged under more urbanized (i.e., warmer and drier) conditions.

Finally, as the mobility of caterpillars is limited compared to adult mobility, local conditions should be predominant in determining individual performance. We hence predict urbanization at the small scale (200 × 200 m subplots), rather than at the large scale (3 × 3 km plots), to affect butterfly fitness traits.

## Methods

### Study species

The Speckled wood (*P. aegeria* L.) and the Wall brown (*L. megera* L.) are closely related Palearctic satyrine butterflies. Both species use several grass species as host plants on which they lay their eggs singly (Wiklund 1984). In Europe, the two species differ in conservation status: *P. aegeria* is abundant in most of its range, which has extended northwards over the last decades (Pateman et al. 2016), whereas *L. megera* shows (severe) declines in several countries, especially in NW‐Europe (Van Dyck et al. 2015).

### Study sites

**Figure 1:**
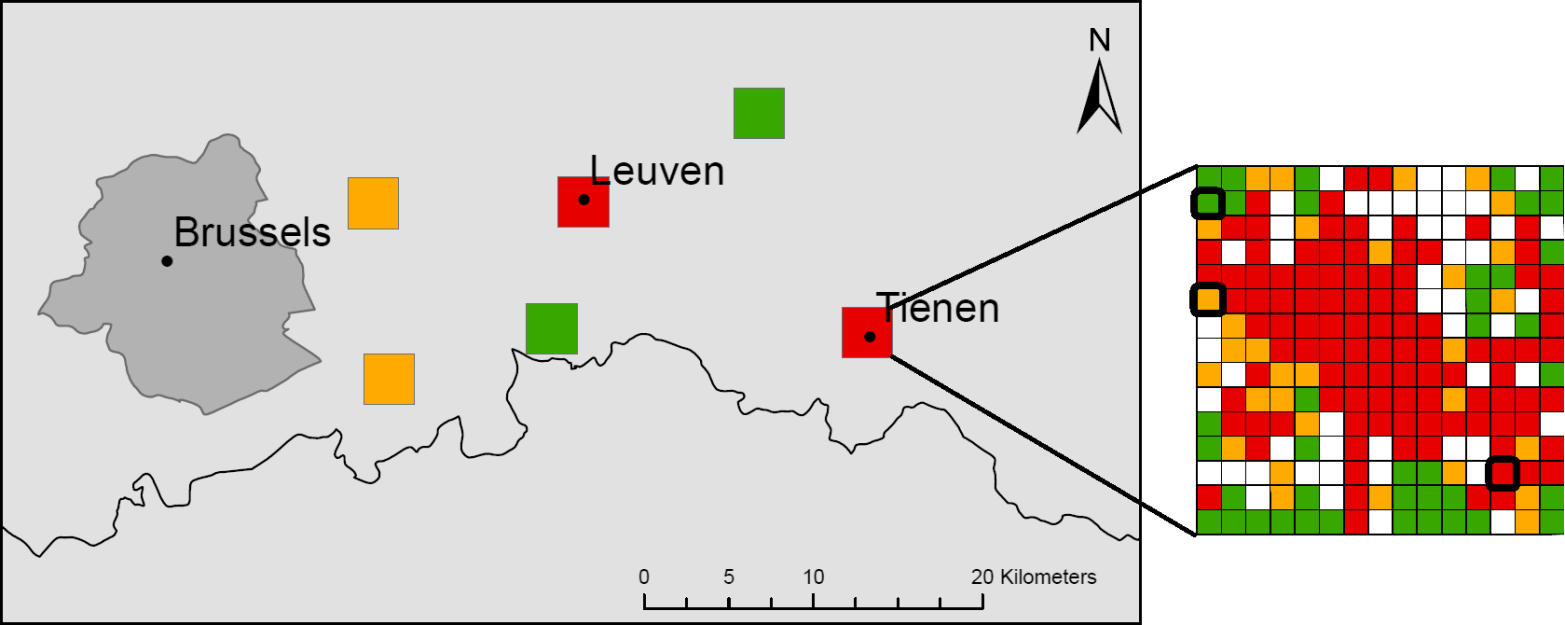
Map of the study area in central Belgium depicting the six plots. On the right, an example of the different subplots within a given plot, highlighting the three selected subplots in bold. Rural, semiurban, and urban categories are depicted in green, orange, and red, respectively, with white subplots depicting urbanization degrees that fall outside these three urbanization classes. This figure depicts the two spatial scales (i.e., 200 × 200 m and 3 × 3 km) at which urbanization effects were investigated on climatic variables and on the biological fitness of two butterfly species.

We selected sites following the same procedure as Tüzün et al. (2015). Using a vectorial layer with the precise contours of all buildings (i.e., the Large‐scale Reference Database, an object‐oriented reference map of Flanders https://www.agiv.be/international/en/products/grb-en) in a GIS, we selected six 3 × 3 km study plots in central Belgium covering an urbanization gradient at the landscape scale: two urban plots (built‐up area > 15%), two semi‐urban plots (built‐up area 5–10%), and two rural plots (built‐up area < 3%). Each plot was then further divided in 200 × 200 m subplots. The same levels of percentage built‐up area applied at the plot scale to delineate the three urbanization classes at the landscape scale were also applied at the subplot scale. Within each plot, we selected three subplots of different urbanization classes that contained wooded elements. If several suitable subplots were available for a given subplot category, the study subplot was chosen randomly. Together, this combination of plots and subplots hence represents two urbanization gradients: one at the landscape scale and one at the local scale (Fig. 1).

### Split‐brood field experiment

During the summer of 2013, gravid butterfly females were captured in nonurban sites and brought to the laboratory for oviposition in individual cages on the grass *Poa trivialis*. Females of *P. aegeria* were captured at two woodland sites (i.e., Bois de Lauzelle and Hallerbos) and two agricultural sites (i.e., Hoegaarden and Rillaar) in central Belgium between July 23 and August 6. Females of *L. megera* were captured between August 10 and 19 at two sites in south‐east Belgium (i.e., Longvilly and Compogne; agricultural sites dominated by hay meadows and pastures) as the species has recently disappeared from central Belgium. At each one of these six sites, three females were captured. During oviposition, females had access to a cotton soaked in honey water.

From each of the 18 females, we used a minimum of 54 larvae, which we spread over 18 pots per female. Groups of three young (first or early second instar), full‐sib caterpillars were placed within single enclosed host‐plant pots (diameter: 15 cm; height: 30 cm). Pots containing *P. aegeria* larvae were placed on‐site between August 6 and 21, and those with *L. megera* larvae between August 22 and September 3. As we could not mark larvae, we did not mix families within individual enclosures. The host plant used for both species was *P. trivialis*, which were grown from seeds on a standardized soil mixture and under standard conditions in a climate room (16L:8D, 26°C:16°C). Plants were watered every other day. The offspring of each female was then spread equally among the 18 subplots (i.e., one enclosure in each subplot). For each female, three additional enclosures (containing three larvae each) were placed on the site where that female was caught (hereafter: site of origin).

After 1 month on the field, we checked host‐plant condition; each plant was assigned to one of two categories based on its visual appearance: healthy (i.e., dominance of green leaves) or withered (i.e., all or most leaves brown, indicating the plant suffered from drought). We also checked enclosures every 5 days for pupae, which we took to the laboratory. However, some larvae (mostly in their last instar) did not reach the pupal stage by mid‐October. To avoid larvae or pupae entering winter diapause (Van Dyck and Wiklund 2002), these larvae were brought back in the laboratory (16L:8D; 26°C:16°C), so they would still develop directly into adults. As such, time spent on the field ranges from 50 to 65 days for *P. aegeria* and from 43 to 54 days for *L. megera*. Pupae were kept individually in labeled plastic cups until adult emergence under conditions identical to those used for rearing the host plants.

### Microclimatic measurements

Integrated recorders that automatically registered and logged air temperature and relative humidity were placed within 5 m of the pots and 1 m above the ground in each subplot and site of origin (HOBO U23 Prov2 U23‐001). They recorded temperature (accuracy: 0.21°C; resolution: 0.02°C) and relative humidity (accuracy: 2.5%; resolution: 0.03%) every 5 min between August 30 and October 8. Eventually, we derived nine climatic variables from the obtained series: mean temperature, mean daytime and mean night‐time temperature, maximum and minimum temperature, temperature range, mean relative humidity, mean daytime, and mean night‐time relative humidity. These variables were calculated for each day of the study period. To compute daytime variables, data from 1 h after sunrise to 1 h before sunset were used. Similarly, we used data from 1 h after sunset to 1 h before sunrise to compute night‐time variables.

### Fitness measures

Larval survival was defined as the proportion of larvae that reached the pupal stage. At the day of emergence, adults were killed and stored by freezing (−21°C). Afterward, frozen individuals were placed in an incubator at 60°C for 24 h, and dry body mass was measured using a microbalance (Ohaus Explorer; accuracy: ±0.1 mg; repeatability:  > 99%).

### Statistical analyses

Data were analyzed with a two‐step approach. First, we used mixed linear regression models (R‐package *lme4*) to analyze the effect of urbanization on climatic data and fitness traits. Then, we compared these results with data obtained from the sites of origin.

First, each of the nine climatic variables was used as a dependent variable, with plot type, subplot type and their interaction as fixed factors. Subplot type nested within the plot identifier and date (to account for between day variation) were included as random factors. For adult mass, we conducted separate analyses for the two species as their larvae did not spend exactly the same amount of time on the field. We ran models for each sex separately as important sex‐related morphological differences do occur in both species (Wiklund and Karlsson 1988). Fixed effects included plot type and subplot type, and for *P. aegeria* also ecotype (i.e., woodland or agricultural). Family and subplot type (nested within the plot identifier) were included as random factors. Dry mass was log‐transformed to obtain normality. Larval survival could not be discriminated for males and females separately. We used logistic regression and performed likelihood ratio tests. Factors of interest included subplot and plot type, and for *P. aegeria* also ecotype. Family and subplot type (nested within the plot identifier) were included as random factors. For all tested variables, full models included the factors of interest and their two‐way interactions. Final models were obtained using stepwise backward elimination of nonsignificant factors with *P *>* *0.10.

Second, we performed multiple comparisons in order to compare larval survival and adult size between the sites of origin and the different subplot types, adjusting significance levels with the Bonferroni correction (R‐package *multcomp*).

In order to look into climatic effects on the larval survival of *L. megera*, we carried out a principal component analysis (PCA) (R‐package *ade4*), since several of the measured climatic variables were correlated; for each subplot, and for each of the nine climatic variables, values were averaged over the total logging period, and scaled and centered prior to the PCA. Next, we fitted a logistic regression with the PC1 and PC2 coordinates of each subplot (and their interaction) as fixed effects, and family and plot identifier as random effects.

All analyses were carried out with R 3.2.3 (R Core Team 2015).

## Results

### Urban Heat Island

**Figure 2:**
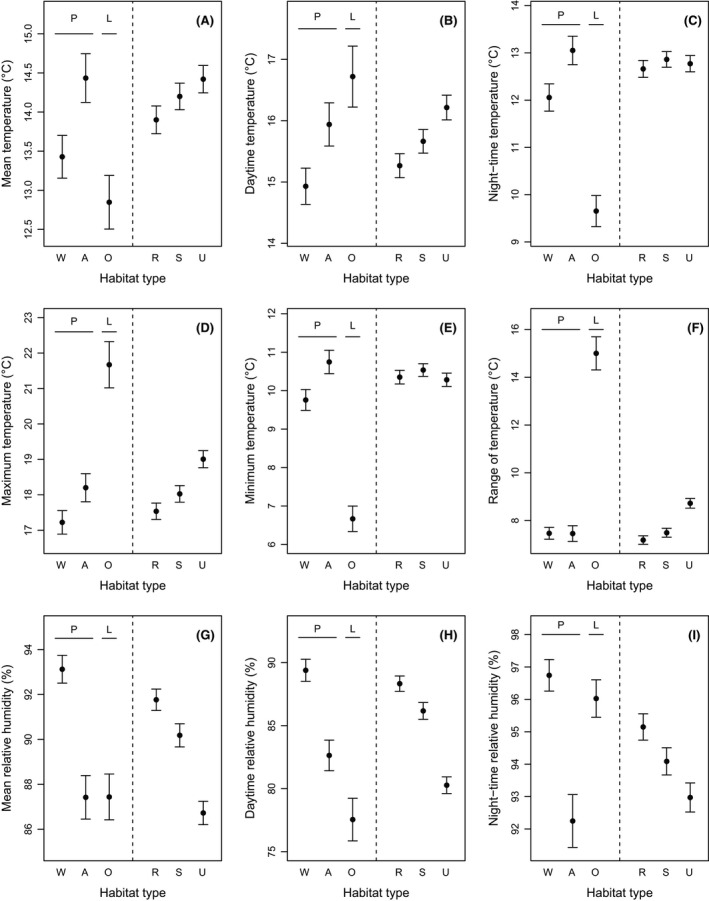
Effect of habitat type on nine climatic variables. On the left of each panel, W, A, and O stand for Woodland, Agricultural, and Open vegetation, respectively. The former two represent the sites of origin (i.e., reference) for the butterfly *P. aegeria* (P) whereas the latter represents the sites of origin for the butterfly *L. megera* (L). On the right of each panel, R, S, and U stand for Rural, Semiurban, and Urban, respectively. These three categories represent different levels of urbanization at the subplot (i.e., local, 200 × 200 m) scale. For each climatic variable, we show mean values (±SE).

Subplot urbanization type affected several climatic parameters (Fig. 2). During the day, urban subplots were on average 0.94°C warmer relative to rural subplots, with a difference in maximum temperature of 1.47°C (*F*_2,12_ = 17.39, *P* < 0.001; *F*_2,12_ = 15.22, *P* < 0.001, respectively; Fig. 2B,D). As a consequence, the temperature range increased with increasing urbanization at the subplot level (+1.54°C between rural and urban subplots; *F*_2,12_ = 10.59, *P* = 0.002; Fig. 2F). Also, urban subplots experienced lower mean relative humidity (−5.04%; *F*_2,12_ = 7.79, *P* = 0.007; Fig. 2G), largely because daytime relative humidity was lower in urban subplots compared to rural subplots (−8.05%; *F*_2,12_ = 11.67, *P* = 0.001; Fig. 2H). Plot type and the plot type × subplot type interaction had no effect on climatic variables (*P *>* *0.05).

After 1 month on‐site, all host plants used for the *L. megera* larvae were still of good quality. For *P. aegeria*, the frequency of host plants in good condition decreased significantly with subplot urbanization type: 86% (67/78) in rural, 76% (59/78) in semi‐urban and 58% (45/77) in urban subplots ($\chi_{2}^{2}$ = 12.69, *P* = 0.001).

### Larval survival

In *P. aegeria*, overall larval survival across the urbanization gradient set‐up was 37% (Table 1). Survival rate was not significantly affected by degree of urbanization at the landscape scale ($\chi_{2}^{2}$ = 3.47, *P* = 0.17), nor at the local scale ($\chi_{2}^{2}$ = 0.51, *P* = 0.91), nor by ecotype ($\chi_{2}^{2}$ = 0.49, *P* = 0.78). Both ecotypes responded similarly to urbanization (i.e., nonsignificant ecotype by plot type and ecotype by subplot type interactions). At the sites of origin, survival was similar for both ecotypes (Post hoc test: *P* = 0.09). Within ecotypes, larval survival at the sites of origin was not better than survival in the urbanization gradient (Post hoc test: *P *>* *0.1).

**Table 1:** Percentages of larval survival in the butterflies *L*. *megera* and *P*. *aegeria* at the sites of origin and in plots and subplots characterized by three levels of urbanization

	*L. megera*	*P. aegeria* – Agricultural ecotype	*P. aegeria* – Woodland ecotype
Sites of origin	72.2 (39/54)	25.9 (14/54)	43.1 (22/51)
Subplot type			
Rural	31.5 (34/108)	41.0 (48/117)	36.7 (43/117)
Semiurban	45.4 (49/108)	29.9 (35/117)	41.9 (49/117)
Urban	62.7 (64/102)	35.1 (40/114)	35.0 (41/117)
Plot type			
Rural	57.4 (62/108)	39.3 (46/117)	48.7 (57/117)
Semiurban	31.4 (32/102)	30.7 (35/114)	28.2 (33/117)
Urban	49.1 (53/108)	35.9 (42/117)	36.7 (43/117)

In *L. megera*, overall larval survival across the urbanization gradient set‐up was 46% (Table 1). Survival rate increased significantly with degree of urbanization at the subplot level ($\chi_{2}^{2}$ = 7.14, *P* = 0.028). Survival was also affected by the degree of urbanization at the plot level ($\chi_{2}^{2}$ = 6.67, *P* = 0.035) and was the lowest in semiurban plots. Survival at the sites of origin was significantly higher than in rural (Post hoc test: *P* = 0.008) and semiurban subplots (Post hoc test: *P* = 0.008) but did not differ significantly from survival in urban subplots (Post hoc test: *P* = 0.98).

Having shown significant effects of urbanization on larval survival in *L. megera*, we went on to test to what degree its survival is explained by the tested climatic variables using a PCA. This PCA generated two principal components with eigenvalues greater than one, explaining together 96.6% of the variance. PC1 was positively correlated with mean, daytime and maximum temperatures, but negatively correlated with mean, daytime and night‐time relative humidity. PC2 was positively correlated with the range of temperature, but negatively correlated with night‐time and minimum temperature (Table S1). In *L. megera*, larval survival increased with increasing values of both PC1 and PC2 (estimate ±1 SE: 0.191 ± 0.056, $\chi_{1}^{2}$ = 11.57, *P* = 0.0006; 0.278 ± 0.089, $\chi_{1}^{2}$ = 9.67, *P* = 0.002, respectively) (Fig. S1).

### Size at reproduction

Male *P. aegeria* (*N* = 124) that developed in semiurban subplots had a lower total dry mass (*F*_2,111.8_ = 5.21, *P* = 0.006 – Table 2) compared to males of the other subplot types, with males of the agricultural ecotype tending to respond more strongly (ecotype × subplot type: *F*_2,111.2_ = 2.68, *P* = 0.07). Body mass did not vary with urbanization at the landscape scale (*F*_2,111_ = 0.88, *P* = 0.41). In females (*N* = 110), there were no significant differences for this trait relative to the degree of urbanization at the local scale (*F*_2,10.9_ = 1.19, *P* = 0.34) nor at the landscape scale (*F*_2,11.3_ = 2.59, *P* = 0.11). Butterflies of the agricultural ecotype that developed at the sites of origin (*N* = 14) weighed less than agricultural adults that developed in rural and urban subplots (Post hoc tests: *P* = 0.006 and *P* = 0.017, respectively – Table 2). Contrary to the agricultural ecotype, butterflies of the woodland ecotype had a similar dry mass at the sites of origin (*N* = 22) and in the urbanization set‐up (all Post hoc tests: *P *>* *0.05).

**Table 2:** Total dry mass (mg; mean ± SE) of male and female *L. megera* and *P. aegeria* butterflies that developed at the sites of origin (i.e., reference) and in subplots (200 × 200 m) characterized by three levels of urbanization. The number of measured individuals (*N*) is indicated between brackets

	*L. megera*	*P. aegeria* – Agricultural ecotype	*P. aegeria* – Woodland ecotype
Males			
Sites of origin	22.2 ± 0.7 (*N* = 20)	12.6 ± 1.4 (*N* = 6)	14.8 ± 1.0 (*N* = 9)
Rural	17.2 ± 0.9 (*N* = 18)	16.7 ± 0.7 (*N* = 29)	15.6 ± 0.8 (*N* = 24)
Semiurban	17.0 ± 0.9 (*N* = 27)	12.9 ± 1.1 (*N* = 13)	15.2 ± 0.6 (*N* = 21)
Urban	19.4 ± 0.6 (*N* = 33)	15.9 ± 0.7 (*N* = 24)	16.8 ± 1.2 (*N* = 14)
Females			
Sites of origin	32.3 ± 1.1 (*N* = 13)	18.1 ± 1.7 (*N* = 8)	19.7 ± 1.1 (*N* = 13)
Rural	25.9 ± 1.4 (*N* = 12)	22.8 ± 1.0 (*N* = 16)	22.4 ± 1.1 (*N* = 17)
Semiurban	28.1 ± 1.1 (*N* = 17)	20.7 ± 1.1 (*N* = 17)	20.7 ± 1.1 (*N* = 22)
Urban	28.0 ± 1.2 (*N* = 24)	22.9 ± 0.9 (*N* = 14)	20.6 ± 1.0 (*N* = 24)

In *L. megera*, urbanization at the landscape scale had no effect on total body mass, neither in males (*N* = 78, *F*_2,5.9_ = 1.64, *P* = 0.27) nor females (*N* = 53, *F*_2,13.1_ = 0.48, *P* = 0.63). However, males tended to develop a larger body size with increasing urbanization at the local scale (*F*_2,13.3_ = 2.95, *P* = 0.08 – Table 2), while females showed no such pattern (*F*_2,5.2_ = 0.43, *P* = 0.67). Adults that developed at the sites of origin (*N* = 34) reached a higher body mass than individuals reared at rural (Post hoc test: *P* = 0.02) and semiurban subplots (Post hoc test: *P* = 0.01), but had a similar body mass to urban subplot butterflies (Post hoc test: *P* = 0.25).

## Discussion

Using an in situ experimental approach with a split‐brood design, we showed that urbanization affects larval microclimates, and consequently, larval survival and adult butterfly size/mass. These effects of urbanization were only significant when considering urbanization at the local scale. Hence, we argue that studies in the context of urbanization, and more widely in the context of human‐induced rapid environmental change, should also focus on fine‐grained spatial scales for insects, and probably for several other organisms. We discuss our results in terms of larval pre‐adaptations to city life conditions and develop perspectives for future research on the interactions between urbanization and climate change.

### The urban microclimate and its implications

The UHI is considered as an important ecological consequence of urbanization (Shochat et al. 2006). It occurs over large spatial extent (e.g., Streutker 2003) and its ecological footprint can extend well beyond the city limits (Zhang et al. 2004). Here, we showed that this concept also applies to microhabitats relevant for the development and activity of ectotherm larvae in or near wooded elements; urbanization measured at the local scale (200 × 200 m) was the only factor to influence woodland microclimate: urban subplots were warmer and drier during the day and experienced higher maximum temperatures and increased within‐day variation in temperature than their rural counterparts. Surprisingly, the UHI disappeared at night, although it has predominantly been reported to be a nocturnal phenomenon (Oke 1982; Arnfield 2003). Historically, the UHI was mainly perceived at a coarse scale and was estimated by comparing pairs of stations in open habitats, typically downtown versus airport sites (Bohm 1998). Instead, we monitored climate at a fine spatial scale (i.e., almost at the level of the host plant), in woodland elements whose microclimate often differs from nearby open locations (Suggitt et al. 2011). In particular, the buffering effect provided by the forested vegetation may explain why we found overall small differences in temperature (of about 1°C) compared to previous work on the UHI (e.g., up to 12°C; Klysik and Fortuniak 1999). We thus suggest that open vegetation types – and their associated community of living organisms – are likely to be affected to a greater extent by the UHI. The interest of the UHI effect relative to microhabitats relevant to small organisms is only recent. For instance, Vermunt et al. (2012) measured under‐bark temperatures and reported the UHI in this microhabitat to be only 0.4°C. To date, most species distribution models deal with data whose spatial resolution is on average more than 1000 times larger than the organism concerned (Potter et al. 2013). Coarse scale data, however, are unlikely to fully capture small‐scale environmental heterogeneity (Randin et al. 2009). This may be particularly true for small climatic differences driven by habitat types. Because urbanization has a strong impact on biodiversity and its significance will further increase (Alberti 2015), more studies should focus on quantifying the UHI at spatial scales relevant for the studied organisms, including small spatial scales for several insects that cover much taxonomic richness and aspects of ecosystem functioning (e.g., pollination). Such information is currently often lacking.

### Urbanization and butterfly fitness traits

Urbanization not only affected microclimate, but also butterfly fitness‐related traits. In line with our hypothesis that habitat of origin drives a species' response to urbanization, larval survival increased steadily along the urbanization gradient, and urban subplots (compared to rural and semiurban subplots) tended to result in larger adult males in the thermophilous butterfly *L. megera*. Urbanization at the subplot scale (i.e., 200 × 200 m), rather than at the plot scale (i.e., 3 × 3 km), explained this variation in fitness traits, consistent with our idea that caterpillars respond predominantly to local conditions. Twice as many larvae reached the pupal stage in urban compared to rural subplots, and the biggest adult males were produced in urban subplots too. Although the sites of origin had the highest larval survival rates and produced the heaviest adults, differences with urban subplots were not significant. As a result, we conclude that, along an urbanization gradient, this thermophilous species directly benefits from developing under urban microclimatic conditions. This result is probably relevant for thermophilous species in general.

*Lasiommata megera*'s benefits in terms of larval survival and adult body mass within urban sites are most likely explained by urbanization‐mediated effects on microclimate. Lowe et al. (2014) too suggested increased temperature and prey availability in urban areas to explain the increased body size and fecundity with increasing urbanization at multiple spatial scales in wild‐caught individuals of the orb‐weaving spider *Nephila plumipes*. Our split‐brood experiment, over a gradient of urbanization, showed that *L. megera* larval survival is improved under dry, warm daytime conditions with high maximum temperatures as well as in sites with a large within‐day variation in temperature and low minimum and night‐time temperatures. Interestingly, most of these conditions are met in urban subplots, but especially so at the sites of origin (Fig. 2). We thus explain the positive impact of urban conditions on larval survival and adult body mass of *L. megera* by urban subplots locally providing climatic conditions which are more similar to the natural habitat of the species.

Temperature effects have attracted much attention in studies dealing with the life history and developmental biology of insects and there is a straightforward link with the UHI framework. However, far fewer studies have addressed the role of relative humidity. Our results are congruent with earlier work on the resistance to desiccation in the same study species (among other satyrine butterflies) at the egg and young larval stage; resistance to desiccation under low relative humidity was higher in *L. megera* of warm and dry natural habitats compared to *P. aegeria* of humid woodland habitat (Karlsson and Wiklund 1985). Urban areas are likely to increase dehydration stress at different life stages and resistance can be accomplished through different physiological mechanisms (Gibbs and Matzkin 2001). Urban subplots were as dry as *P. aegeria* habitat in agricultural areas, well below the humidity level of woodland (Fig. 2G). Further work on the water balance of butterflies in urban areas, as well as in other habitats, is now warranted, including for example the significance of (compensatory) nectar exploitation in adults (Winkler et al. 2009).

Besides their direct effects on the butterflies, both temperature and relative humidity may also affect butterfly development by modifying host‐plant quality. We used host plants reared under common garden conditions, and although all plants appeared of good quality after 1 month on the field, we cannot exclude that the high temperatures and reduced relative humidity experienced in urban subplots may have triggered changes in leaf micronutrients. In butterflies, both negative and positive impacts of foraging on stressed host plants have been documented (e.g., Bauerfeind and Fischer 2013a, 2013b). Thus, both direct and indirect effects of temperature and relative humidity may be involved in the urbanization‐related developmental plasticity of *L. megera*.

Unlike for *L. megera*, urban conditions had little effect on *P. aegeria*. Larval survival did not differ along the urbanization gradients at both spatial scales, and was similar to survival at the reference sites of origin. This suggests that larvae of this species can tolerate a wide range of environmental conditions, independent of the ecotype. Hence, from a development point of view, it has a better ability to buffer against environmental variation. Urbanization had no impact on adult body mass in females, but – unexpectedly – males were smaller in semiurban subplots. Temperature and relative humidity are unlikely to play a role here as none of the nine climatic variables showed peak levels at intermediate levels of urbanization. Although we cannot provide any clear explanation to the reduced size of semiurban butterflies, it is worth noting that the pattern tended to be more pronounced in males of an agricultural landscape origin. Increased environmental variability is expected to favor increased plasticity (De Jong 1995). Because temperature and wind speed are more variable in agricultural landscapes (Merckx et al. 2008), the agricultural ecotype of *P. aegeria* is hence likely to have evolved toward increased levels of phenotypic plasticity. In line with this prediction, Vandewoestijne and Van Dyck (2011) found that the agricultural ecotype displayed steeper latitudinal clines for flight‐related morphological traits, including body mass. The consequences of increased developmental plasticity in a context of urbanization warrants further investigation.

It is also worth noting that larval survival and adult body mass were highest at the sites of origin for *L. megera*, whereas for *P. aegeria* fitness was similar – sometimes lower – at the sites of origin compared to the experimental sites. This may well suggest local adaptation to specific microclimatic conditions for *L. megera*, whereas *P. aegeria* seems more tolerant to variation in temperature and/or relative humidity (at least under the range of conditions experienced here by the larvae).

### What defines an urban exploiter among insects?

Insects have complex life cycles, and different life stages can be affected differently by urbanization. Our experiment allows to disentangle pre‐adaptations to urbanization at the early life stages from the adult stage. Based on our results, we suggest two traits as potential larval pre‐adaptations to the successful colonization of urban areas by insects.

Adverse conditions are particularly challenging for invertebrate larvae. In most species, the larval stage has limited mobility. Hence, the ability of larvae to escape harsh conditions is reduced. Because of evolutionary adaptation to warm, dry conditions, we suggest that thermophilous species are better prepared to cope with urban conditions. In particular, thermophilous insects and their larvae may be better armed to face the higher desiccation risks at urban sites, in line with the associated lower relative humidity levels of the air and more frequent extreme temperature events. Here, we consider thermophily in a broad sense; referring to native warm‐adapted species, but also to species or individuals originating from warmer latitudes. Because urban areas typically provide climatic conditions similar to warmer latitudes, organisms associated with these warmer latitudes are likely to better handle the warmer and drier urban conditions, although they cannot be termed as thermophilous sensu stricto. A complementary larval pre‐adaptation to survive urban conditions is to display a broad environmental tolerance. This trait has been proposed as a predisposition for birds to thrive in human‐dominated habitats (Bonier et al. 2007), but we suggest this is a more general phenomenon that applies to invertebrates as well.

Given the complex life cycles in holometabolous insects, we are aware that larval fitness in urban environments may not necessarily reflect biological success at the adult stage. *L. megera* is a clear example of such a disconnection among life stages. Although we found larvae of this species to survive better under urban conditions than under rural and semiurban conditions, the species has recently declined in N.W. Europe (Van Dyck et al. 2015). Moreover, it is an uncommon species in urban areas, at least at temperate latitudes (Bergerot et al. 2011). Although temperature is indeed a key parameter for larval development and adult activity in ectotherms, other factors too will affect the biological success (Dennis et al. 2003). Provision of nectar quantity and quality, for example, is likely to be different in urban areas as a consequence of altered plant communities (Baldock et al. 2015). This may well be an important issue for income breeders whose reproductive output is ultimately determined by the amount of food resources they can find as adults (e.g., Javois et al. 2011). Also, suitable urban habitat patches (e.g., brownfields, wastelands) are often characterized by high disturbance levels and turn‐over rates, with interpatch distances additionally likely to exceed the dispersal capacities of *L. megera*, preventing the species from establishing and maintaining viable populations in urban areas. More generally, the ability to pick up informative environmental cues to make appropriate developmental decisions has been suggested to be important in a context of rapid environmental change (Van Dyck 2012). As urban areas typically provide warmer and drier conditions compared to rural locations at the same latitude, this ability might be significant for invertebrates to persist in cities after colonization. Thus, larval pre‐adaptations alone are unlikely to determine whether an insect species is an urban exploiter. Instead, these pre‐adaptations should be coupled with a suite of adult life‐history traits (e.g., mobility and diet; Concepción et al. 2015) that make a species successful for urban life. There is a need for studies assessing the response to urbanization throughout the complete life cycle of organisms like insects. Such integrated studies would help in getting a more realistic view on the impact of urbanization on biodiversity.

### Perspectives

In recent years, much has been done to improve biodiversity in cities and there is evidence that small patches of seminatural vegetation can be of conservation value, providing habitat to rare and regionally declining species (e.g., Öckinger et al. 2009; Baldock et al. 2015). Our results show that the effect of urbanization on climate acts at the local scale, which is of conservation significance as a given landscape should hence not be considered as thermally homogeneous, but rather as a mosaic of cold and warm spots. In an urbanization context, this thermal heterogeneity caused by the local‐scale configuration of build‐up and interspersed open locations may provide refugia to escape from the UHI. Such local “cold islands” may be of particular importance for cold‐adapted species that may be especially sensitive to stressful urban thermal conditions. Assuming that these patches are functionally connected and large enough to sustain viable (meta‐) populations, they could be important elements allowing species to persist under harsh conditions even in intensively managed urban landscapes.

Cities have also recently been acknowledged as an appropriate, but still underused study system for climate change research (Youngsteadt et al. 2015). Urbanization and climate change are two elements of global change, and likely to interact with each other. The global average air temperature is expected to rise by up to 4°C by 2100 (IPCC, 2014). The effect of a concomitant increase in temperature due to the UHI and global warming on biological systems is unknown, although a recent study suggests that their combined effect is not simply additive (Diamond et al. 2014). Moreover, a rise in average temperature leads to a much higher frequency of extreme temperature and drought events. Over the last decade, the likelihood of extremely hot summers has risen by a factor 10 (Christidis et al. 2015) and future heat waves are expected to last longer and to be more severe (Schoetter et al. 2015). Synergies between urban and global warming will result in organisms to be more frequently exposed to extreme climatic conditions. The effect of the latter might be more important than an increase in average conditions (Bauerfeind and Fischer 2014), especially for species that already live close to their upper thermal limit (Nguyen et al. 2011). Butterflies are highly suitable organisms for addressing these issues in a biogeographic context through split‐brood experiments, as we used here.

Based on our fine‐grained effects of urbanization on biological fitness components and fitness‐related traits in ectotherms, we call for more research that would focus on integrating fine‐grained effects of urbanization with other aspects of global change. Although this kind of research may be technically challenging, an integrated research framework would give a much better, and much needed insight into the combined effects of multiple human‐induced rapid environmental changes on biodiversity.

## Data Accessibility

Data are available from the Dryad Digital Repository: http://dx.doi.org/10.5061/dryad.8s835.

## Conflict of Interest

None declared.

## Supporting information


**Table S1.** Results of the principal component analyses on nine climatic variables.
**Figure S1.** Relationship between larval survival in *L. megera* and the principal components.
