# Supplementary material for: The Urban Heat Island and its spatial scale dependent impact on survival and development in butterflies of different thermal sensitivity
Source: Ecol Evol. 2016 May 21;6(12):4129–40. doi: 10.1002/ece3.2166 (PMC4972237; doi:10.1002/ece3.2166)

**Supporting Information**

**Table S1:** Results of the principal component analyses on nine climatic variables. Loadings of each variable are provided for the first two principal component axes, which are characterised by eigenvalues >1.

|  | PC1 | PC2 |
| --- | --- | --- |
| Mean temperature | 0.9333867 | -0.3332216 |
| Daytime temperature | 0.9251592 | 0.3109366 |
| Night-time temperature | 0.5787494 | -0.8085110 |
| Maximum temperature | 0.8742866 | 0.4084711 |
| Minimum temperature | 0.4236879 | -0.8954551 |
| Range of temperature | 0.4217485 | 0.8978095 |
| Mean relative humidity | -0.9748814 | -0.1290028 |
| Daytime relative humidity | -0.9283522 | -0.3266489 |
| Night-time relative humidity | -0.9176999 | 0.3204760 |
| Variance explained | 64.8% | 31.8% |

**Figure S1**: Relationship between larval survival in *L. megera* and PC1 (a) and PC2 (b). Correlations between the principal component and the climatic variables are available in Table S1. Regression lines are built with back-transformed (inverse logit) coefficients from the logistic regression model.


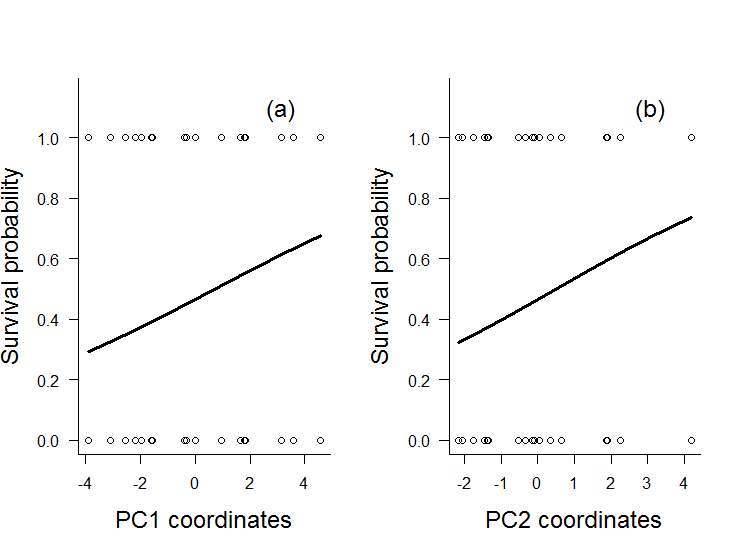

Supplement: Supplementary file 1 — Table S1. Results of the principal component analyses on nine climatic variables. Figure S1. Relationship between larval survival in L. megera and the principal components. [file ECE3-6-4129-s001.docx]
